# Ninjurin1 positively regulates osteoclast development by enhancing the survival of prefusion osteoclasts

**DOI:** 10.1038/s12276-018-0201-3

**Published:** 2019-01-16

**Authors:** Sung-Jin Bae, Min Wook Shin, Taekwon Son, Hye Shin Lee, Ji Soo Chae, Sejin Jeon, Goo Taeg Oh, Kyu-Won Kim

**Affiliations:** 1 College of Pharmacy and Research Institute of Pharmaceutical Sciences, Seoul National University, Seoul, 08826 Korea; 2 Department of Life Sciences and Technology, PerkinElmer, Seoul, 06702 Korea; 3 Department of Life Sciences, Ewha Womans University, Seoul, 03760 Korea; 4 Crop Biotechnology Institute, GreenBio Science and Technology, Seoul National University, Pyeongchang, 25354 Korea; 5 Present Address: Korean Medicine Research Center for Healthy Aging, Pusan National University, Yangsan, 50612 Korea; 6 Present Address: RNA Therapeutics Institute, University of Massachusetts Medical School, Worcester, MA USA

**Keywords:** Apoptosis, Bone development

## Abstract

Osteoclasts (OCs) are bone-resorbing cells that originate from hematopoietic stem cells and develop through the fusion of mononuclear myeloid precursors. Dysregulation of OC development causes bone disorders such as osteopetrosis, osteoporosis, and rheumatoid arthritis. Although the molecular mechanisms underlying osteoclastogenesis have been well established, the means by which OCs maintain their survival during OC development remain unknown. We found that Ninjurin1 (Ninj1) expression is dynamically regulated during osteoclastogenesis and that *Ninj1*^−/−^ mice exhibit increased trabecular bone volume owing to impaired OC development. Ninj1 deficiency did not alter OC differentiation, transmigration, fusion, or actin ring formation but increased Caspase-9-dependent intrinsic apoptosis in prefusion OCs (preOCs). Overexpression of Ninj1 enhanced the survival of mouse macrophage/preOC RAW264.7 cells in osteoclastogenic culture, suggesting that Ninj1 is important for the survival of preOCs. Finally, analysis of publicly available microarray data sets revealed a potent correlation between high *NINJ1* expression and destructive bone disorders in humans. Our data indicate that Ninj1 plays an important role in bone homeostasis by enhancing the survival of preOCs.

## Bone development: Regulating the remodelers

A protein called ninjurin1 regulates the activity of bone-degrading osteoclast cells, and may play an important role in various skeletal degenerative disorders. Normal skeletal development requires careful coordination by bone-building osteoblasts and by osteoclasts, which break down and remodel bone. Researchers led by Kyu-Won Kim of Seoul National University in South Korea have shown that ninjurin1 regulates both the development and survival of osteoclasts. They generated genetically modified mice that lack this protein, and observed notable skeletal defects, including abnormal accumulation of bone mass in long bones such as the femur. Elevated ninjurin1 levels are a feature of conditions such as osteoporosis and rheumatoid arthritis, and the researchers propose that therapeutic agents that target this molecule could help control the bone damage caused by improperly regulated osteoclasts.

## Introduction

Bone is a dynamic tissue maintained by continuous remodeling dependent upon the balance between bone formation by osteoblasts (OBs) and bone resorption by osteoclasts (OCs)^1^. Osteoclasts originate from monocyte/macrophage precursor cells of myeloid lineage^2^. In the bone microenvironment, OC differentiation is mediated by two essential cytokines, macrophage colony-stimulating factor (M-CSF) and receptor activator of nuclear factor κB ligand (RANKL), produced by OBs, stromal cells, and T lymphocytes^3^. M-CSF sustains the survival of OC precursors and mature OCs, and RANKL promotes OC differentiation via the induction of NFATc1, a master transcription factor of osteoclastogenesis, including differentiation, fusion, maturation, activation, and survival^2–5^. Consequently, OCs exhibit unique morphological features, such as multinucleation, which is accomplished by the fusion of mononuclear OC precursor cells, and an actin ring cytoskeleton composed of podosome belts^6,7^. Therefore, fine movements and well-organized actin ring cytoskeletons are prerequisites not only for cell–cell fusion but also for OC resorptive function^7^. Dysregulation of OC development and/or function leads to pathological bone disorders, such as osteopetrosis, Paget’s disease, rheumatoid arthritis, and postmenopausal osteoporosis^8–11^.

Ninjurin1 (Ninj1) is a transmembrane protein identified in neurons and Schwann cells after sciatic nerve injury^12^. We previously reported that Ninj1 is expressed in perivascular macrophages near hyaloid vessels and induces vascular endothelial cell apoptosis via enhanced Angiopoietin2 and Wnt7b secretion^13^. We further reported that Ninj1 is expressed in myeloid cells, such as monocytes, macrophages, and microglia, in experimental autoimmune encephalopathy induced in rat brain and mouse spinal cord and subsequently modulates inflammatory myeloid cell infiltration^14^. Ninj1 is also expressed in monocytes and dendritic cells located in human brain multiple sclerosis lesions, suggesting that the function of Ninj1 is conserved in mice and humans^15^. Additionally, Ninj1 is upregulated by lipopolysaccharide (LPS) and regulates LPS-induced inflammation in macrophages^16,17^.

In contrast, the role of Ninj1 in OCs, which are myeloid lineage descendants, has not yet been elucidated. Here, we found that Ninj1 is expressed in OCs and is dynamically regulated during OC differentiation. *Ninj1*^−/−^ mice show increased trabecular bone volume related to defective OC development; additionally, Ninj1 deficiency leads to increased apoptosis in prefusion OCs (preOCs), resulting in a reduced number of mature OCs. Furthermore, high *NINJ1* expression is associated with human bone disorders, such as rheumatoid arthritis and postmenopausal osteoporosis. Our findings suggest that Ninj1 has a novel role in OC development and bone homeostasis and might represent a potent therapeutic target for destructive bone disorders.

## Materials and methods

### Reagents

Recombinant mouse M-CSF, GM-CSF, IL-4, and RANKL were purchased from BioLegend (San Diego, CA). AF488-phalloidin was purchased from Invitrogen (Carlsbad, CA). Antibodies for immunoblot assays were obtained against NFATc1 (Pierce, Rockford, IL); Oscar (R&D Systems, Minneapolis, MN); TRAP (BioLegend); Integrin β3; c-Fos; cleaved Caspase-3, -8, and -9; and cleaved PARP (Cell Signaling Technology, Mountain View, CA). The previously described anti-Ninj1 Ab_1-15_^18^ was used for immunoblotting assays. For FACS analysis, Fc block, and PE-anti-mouse CD115, APC-anti-mouse CD117, and V450-anti-mouse CD11b antibodies were purchased from BD Biosciences (Bedford, MA), and biotin-anti-RANK antibody and APC/Cy7 streptavidin were obtained from BioLegend.

### Mice and bone analysis

*Ninj1*^−/−^ mice were described previously^18^. All animal studies were performed with male mice to exclude sex hormonal effects. Bone marrow cells were obtained from femurs and tibias of 7-week-old *Ninj1*^−/−^ mice or their wild-type (WT) littermates and used for differentiation into macrophages or OCs. Hind limbs isolated from 6- or 12-week-old mice were scanned individually with a radiology apparatus. Plain radiologic and μCT images were obtained with an IVIS Spectrum CT (PerkinElmer, Waltham, MA) and Quantum GX μCT (PerkinElmer) at 10-μm isotropic resolution, respectively. Trabecular bone samples with lengths of over 1 mm encompassing the region of the distal metaphysis away from the proximal edge of the growth plate in the distal end of femurs were analyzed using Inveon Research Workplace (Siemens, Malvern, PA).

All mice were housed in the animal care facility of Seoul National University under specific pathogen-free conditions, and all animal procedures were approved by the Institutional Animal Care and Use Committees of Seoul National University.

### In vitro osteoclastogenesis

Bone marrow-derived macrophages (BMMs) were obtained by bone marrow cell culture. Briefly, bone marrow cells were isolated by flushing the diaphysis of femurs and tibias with phosphate-buffered saline (PBS) and incubated overnight in α-modified Eagle’s medium (MEM) supplemented with 10% fetal bovine serum (FBS) to remove nonhematopoietic lineage cells. Floating cells were further incubated in α-MEM supplemented with 10% FBS and M-CSF (30 ng/mL). After 3 days, nonadherent cells were removed, and BMMs were used as OC precursors. For OC generation, BMMs were further cultured in α-MEM supplemented with 10% FBS and M-CSF (30 ng/mL) and RANKL (100 ng/mL) for the indicated days. Mouse macrophage/preOC RAW264.7 cells were purchased from the Korean Cell Line Bank (Seoul, Korea) and maintained in Dulbecco’s MEM with 10% FBS. For OC differentiation, RAW264.7 cells were cultured in α-MEM supplemented with 10% FBS and RANKL (30 ng/mL).

### TRAP activity staining and measurement of TRAP activity

Tartrate-resistant acid phosphatase (TRAP) activity was assessed using a TRAP Staining Kit (Kamiya Biomedical Company, Tukwila, WA) according to the manufacturer’s protocol. Following colorization, TRAP-positive multinucleated cells (≥3 nuclei) were counted, and images were obtained with an Axiocam MRc on an Axiovert 200 M microscope (Zeiss, Oberkochen, Germany). For TRAP activity measurements in conditioned media, OC culture media were refreshed 24 h prior to collection on the indicated days and immediately centrifuged to remove cell debris, and supernatants were incubated with the chromogenic substrates in tartrate-containing buffer for 3 h at 37 °C. For lysates, cells were lysed in an extraction buffer containing 100 mM Tris pH 8.0, 150 mM NaCl, 1% NP-40, and proteinase inhibitor cocktail (Calbiochem, San Diego, CA). Following incubation, residual activity was inhibited by 4% NaF for 30 min, and absorbance at 540 nm was measured using a microplate reader (Spectra Fluor; Tecan, San Jose, CA).

### FACS analysis

To identify OC progenitor cells, bone marrow cells were incubated with mouse Fc Block (anti-mouse CD16/CD32) in 3% bovine serum albumin (BSA)/PBS for 30 min. The cells were then stained with PE-anti-mouse CD115, APC-anti-mouse CD117, and V450-anti-mouse CD11b antibodies for 1 h and analyzed via FACS. To characterize OC precursors, BMMs were preincubated with mouse Fc Block and streptavidin in 3% BSA/PBS for 30 min. Subsequently, the cells were stained with PE-anti-mouse CD115, V450-anti-mouse CD11b, and biotin-anti-RANK antibodies followed by incubation with APC/Cy7 streptavidin and subjected to FACS analysis. To discriminate apoptotic cells, BMMs cultured with M-CSF and RANKL were collected and stained with FITC-Annexin V and 7AAD on the indicated days with subsequent FACS analysis. All assays were performed using FACSVerse and analyzed with FACSuite 1.0.5 (BD Biosciences).

### Confocal microscopy

To visualize the multinucleated giant cells, BMMs were cultured on glass coverslips with M-CSF (50 ng/mL) + IL-4 (100 ng/mL) or GM-CSF (50 ng/mL) + IL-4 (100 ng/mL). At day 6, cells were washed with PBS and stained with carboxyfluorescein succinimidyl ester (CFSE, 5 μM) for 5 min. Subsequently, cells were fixed with 4% paraformaldehyde and counterstained with Hoechst 33342. To assess preOC transmigration, BMMs were retrovirally transduced with Lifeact-mCherry (see Supplementary materials and methods). Transduced BMMs were selected and further cultured with M-CSF (30 ng/mL) and RANKL (100 ng/mL) for 3 days. Then, the cells were detached and overlaid on a confluent monolayer of GFP-expressing MC3T3-E1 cells. Serial images were stacked over 10- to 14-μm depths in 0.4-μm intervals. To measure actin ring cytoskeletons and OC size, BMMs were cultured on glass coverslips with M-CSF (30 ng/mL) and RANKL (100 ng/mL) for 6 days and fixed with 4% PFA. Following permeabilization with 0.05% Triton X-100, the cells were stained with AF488-phalloidin to visualize F-actin. The area surrounded by the actin ring cytoskeleton was calculated using ImageJ 1.47t (National Institutes of Health, Bethesda, MD). All images, including Z-plane images, were obtained using an LSM 700 confocal microscope (Zeiss) and processed using ZEN 2012 software (Zeiss).

### Microarray analysis

We compiled 4 publicly available gene expression data sets (accession nos. GSE1964, GSE7158, GSE7524 and GSE27390) of human samples from Gene Expression Omnibus (GEO). Raw feature data from 3 data sets (GSE7158, GSE7524, and GSE27390) were normalized using the MAS5 method (R package affy). Dataset GSE1964 was downloaded from GEO as already normalized. The average expression values were calculated for samples. *NINJ1* gene with an absolute fold change of least 1.3 between compared groups was assigned as differentially expressed. To evaluate statistical significance between the compared groups, a *t* test was applied using SigmaPlot version 12.5.0. (Systat Software Inc., San Jose, CA).

### Statistical analysis

The data are presented as the means ± SD and were calculated and analyzed with SigmaPlot 12.5.0. (Systat Software Inc., San Jose, CA). Two-tailed Student’s *t* test was used to determine the significance of differences between two groups. The data in Figs. 4c and 5b were analyzed with a Mann-Whitney *U* test. The data in Fig. 8c, d were analyzed using a paired *t* test and one-tailed Student *t* test, respectively. Differences with *P* < 0.05 were considered significant.

## Results

### Ninj1 deletion enhances trabecular bone volume in mice

**Fig. 1:**
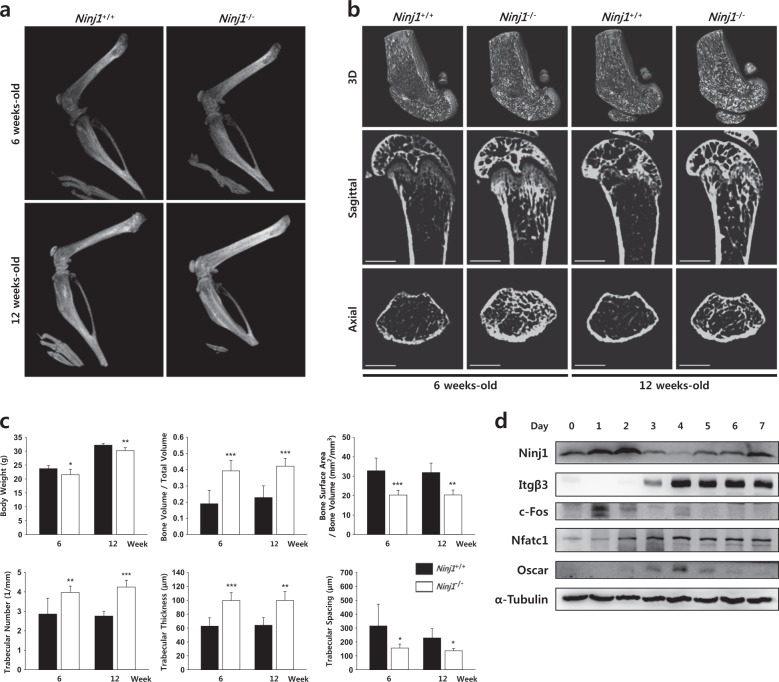
*Ninj1*^−/−^ mice show mild osteopetrosis, and Ninj1 is expressed in osteoclasts. **a** Representative plain X-ray images of hind limbs from *Ninj1*^−/−^ 6- or 12-week-old mice and their corresponding WT littermate mice. **b** Representative μCT images of distal femurs from *Ninj1*^−/−^ 6- or 12-week-old mice and their corresponding WT littermate mice. Scale bars, 1 mm. **c** μCT-determined trabecular bone parameters of distal femurs from 6- or 12-week-old WT (*n* *=* 5, and *n* *=* 4, respectively) and *Ninj1*^−/−^ mice (6-week-old; *n* *=* 7, 12-week-old; *n* *=* 6). **d** Ninj1 expression during osteoclast development. Quantitative data are shown as the mean ± SD, *P < 0.05, **P < 0.01, and ***P < 0.001

We first found that Ninj1 is highly expressed in mature OCs (Supplementary Fig. S1a), suggesting its involvement in bone homeostasis regulation *in vivo*. Assessment of the hind limbs of 6- or 12-week-old *Ninj1*^−/−^ mice by plain radiology indicated that the metaphysis and diaphysis intensities were stronger than those of WT littermates. Furthermore, femur morphologies were linear rather than concave (Fig. 1a). To investigate Ninj1 deficiency-associated bone abnormalities, we compared distal femurs and proximal tibias from 6- or 12-week-old WT and *Ninj1*^−/−^ male mice by μCT analysis. As expected, a clear trabecular bone mass increase was observed in both bones of the *Ninj1*^−/−^ mice (Fig. 1b, Supplementary Fig. S1b). Quantitative measurements revealed increases in bone parameters such as the trabecular tissue volume (Bone Volume/Total Volume), thickness, and number in *Ninj1*^−/−^ mice. In contrast, bone surface area (Bone Surface Area/Bone Volume) and the space between trabecular architectures (Trabecular Spacing) were decreased (Fig. 1c, Supplementary Fig. S1c). These data indicate that Ninj1 deficiency in mice stimulates a mild osteopetrotic phenotype in long bones.

Then, we assessed Ninj1 expression during OC development because in preliminary experiments the expression level of Ninj1 was significantly higher in OCs than in the OB cell line MC3T3-E1 (Supplementary Fig. S2). Ninj1 was upregulated from days 0 to 2, downregulated at days 3 and 4, and restored from days 5 to 7 in BMMs cultured with M-CSF and RANKL (Fig. 1d). In addition, Ninj1 expression in RAW264.7 cells was dose-dependently increased by RANKL (Supplementary Fig. S3a). Collectively, these data suggest that Ninj1 deletion in mice leads to increased trabecular bone volume through modulation of OC development.

### Ninj1 deficiency reduces multinucleated OCs

**Fig. 2:**
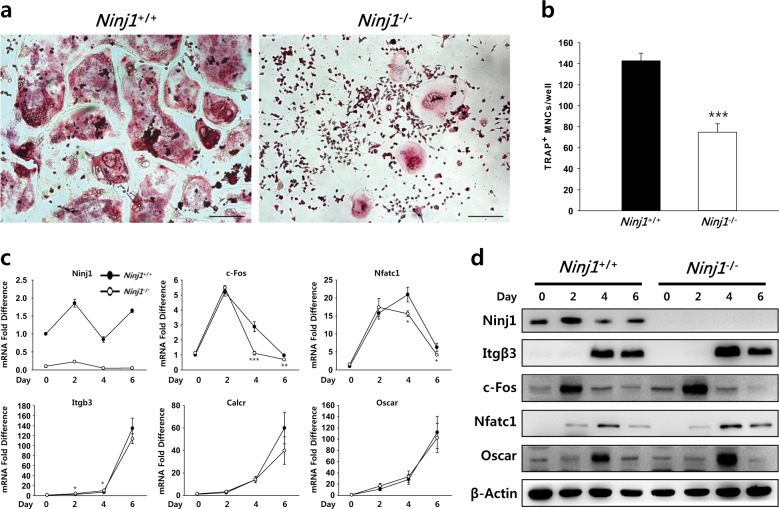
Ninj1 deficiency reduces multinucleated osteoclasts, which is not related to defective differentiation. **a**, **b** WT and *Ninj1*^−/−^ bone marrow cells were cultured with M-CSF for 3 days, and nonadherent cells were discarded. Adherent cells were continuously cultured with M-CSF and RANKL for 7 additional days and subjected to TRAP activity staining. **a** Representative images of TRAP activity staining. Scale bars, 200 μm. **b** Quantitation of TRAP-positive multinucleated osteoclasts. Numbers of TRAP-positive multinucleated osteoclasts (TRAP^+^ MNCs, ≥ 3 nuclei) are shown as the mean ± SD; ***P < 0.001. **c**, **d** The expression of Ninj1 and osteoclast markers during osteoclastogenesis was analyzed by quantitative RT-PCR (**c**) or immunoblot assays (**d**). **c** The mRNA expression of *Ninj1* and osteoclast markers, including *Nfatc1*, *c-Fos*, *Itgb3*, *Oscar*, and *Calcr*, was analyzed, and relative mRNA expression levels are shown as the mean ± SD; *P < 0.05, **P < 0.01, and ***P < 0.001. **d** Protein expression of Ninj1 and osteoclast markers

We then investigated whether the mild osteopetrotic phenotype of *Ninj1*^−/−^ mice represents a defect in OC development. Bone marrow cells from WT and *Ninj1*^−/−^ mice were cultured with M-CSF and RANKL and stained for TRAP activity. TRAP^+^ multinucleated cell number and size were significantly decreased in *Ninj1*^−/−^ cells (Fig. 2a, b). Similar results were obtained in RAW264.7 cells treated with Ninj1 siRNA (Supplementary Fig. S3b–d). Next, we examined typical OC differentiation marker expression. Notably, *Nfatc1* and *c-Fos*, transcription factors for OC differentiation, were unchanged at day 2 and the expression of other OC-specific genes, including *Itgb3*, *Oscar*, and *Calcr*, was comparable in *Ninj1*^−/−^ cells (Fig. 2c, d), suggesting that Ninj1 deficiency disturbs OC development independent of the differentiation process.

### Ninj1 deletion augments OC progenitors

**Fig. 3:**
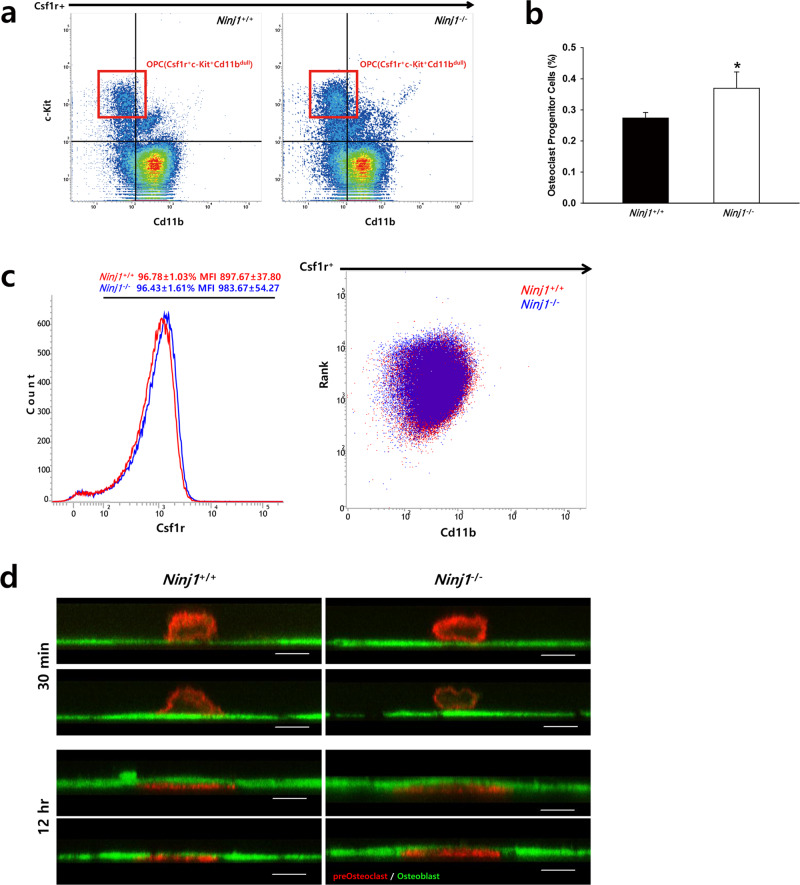
Analysis of osteoclast progenitor cells in the bone marrow, typical marker expression in macrophages, and transmigration of preOCs. **a**, **b** Bone marrow cells were analyzed by FACS. **a** Representative density plots of Csf1r^+^ bone marrow cells. **b** The percent frequency of osteoclast progenitor cells (red box, Csf1r^+^c-Kit^+^Cd11b^dull^) in (A) is shown as the mean ± SD; *P < 0.05. **c** BMMs were analyzed by FACS. The expression of Csf1r is shown in whole cells (left), and Csf1r^+^-gated cells were analyzed for Rank ((y-axis) and Cd11b ((x-axis) expression (right). **d** BMMs transduced with Lifeact-mCherry were cultured with M-CSF and RANKL for 3 days to generate preOCs. Lifeact-mCherry-labeled preOCs were overlaid on a GFP^+^ MC3T3-E1 osteoblast monolayer with the continued presence of osteoclastogenic cytokines. Z-plane images were acquired after 30 min (upper) or 12 h (lower) via confocal microscopy. Scale bars, 10 μm

The proportion of OC progenitors in the bone marrow is important in the early stage of osteoclastogenesis^19^. Because decreased multinucleated OCs might result from reduced progenitor cell numbers, we analyzed the OC progenitor proportion in bone marrow cells from WT and *Ninj1*^−/−^ mice via fluorescence-activated cell sorting (FACS). Csf1r^+^ cells were gated first and further analyzed for c-Kit and Cd11b expression (Fig. 3a). Notably, OC progenitors were 1.37-fold more abundant in *Ninj1*^−/−^ mice (Fig. 3b). The expression of M-CSF receptor and RANK in OC precursors is important for early osteoclastogenesis^19,20^. Thus, we evaluated BMMs, which are used as OC precursors in vitro. FACS and immunoblot analyses indicated that Csf1r, Rank, and Cd11b expression levels were comparable between groups (Fig. 3c, Supplementary Fig. S4).

OCs transmigrate to the OB layer to adhere to mineralized matrix^7^. We previously reported that Ninj1 enhances macrophage transendothelial cell migration^21^. To address this possibility in OCs, we compared the preOC transmigration capabilities^22,23^. BMMs were transduced with Lifeact-mCherry and cultured with M-CSF and RANKL for 3 days to generate mononuclear preOCs. Then, the cells were replaced on a confluent monolayer of OB-like MC3T3-E1 cells expressing GFP. During continuous culture with osteoclastogenic cytokines, Z-plane images were acquired after 30 min or 12 h by confocal microscopy. Both groups of preOCs transmigrated completely through the OB layer and spread well (Fig. 3d). Collectively, these data suggest that Ninj1 is dispensable for OC precursor differentiation and preOC transmigration but might negatively modulate OC progenitor development in mice.

### Macrophage fusion is enhanced by Ninj1 deficiency

**Fig. 4:**
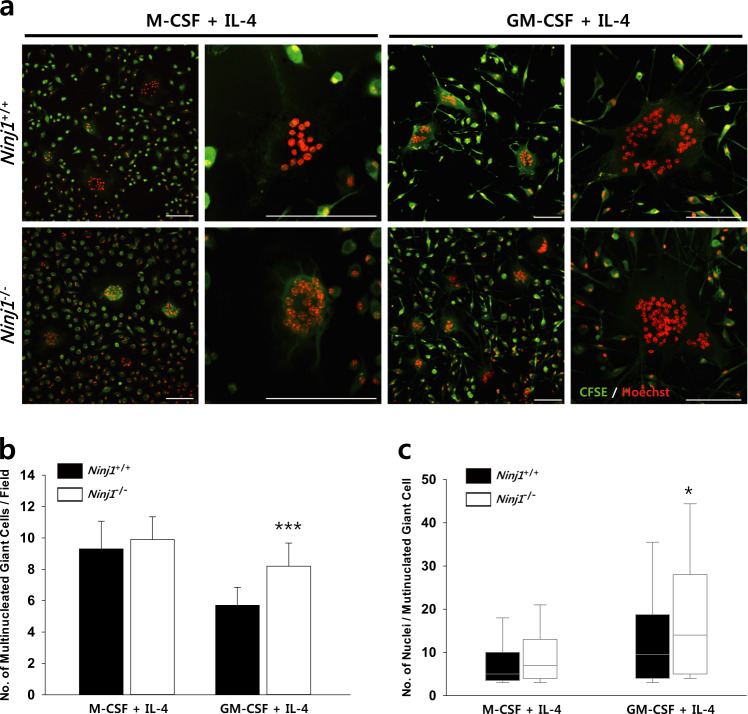
*Ninj1*^−/−^ macrophages generate more multinucleated giant cells. **a**, **c** Multinucleated giant cells were induced by culturing BMMs with M-CSF + IL-4 (left) or GM-CSF + IL-4 (right) followed by staining with CFSE (green) and Hoechst (red). (**a**) Representative images of giant cells. Scale bars, 100 μm. **b** Numbers of multinucleated cells are shown as the mean ± SD; ***P < 0.001. **c** Numbers of nuclei in multinucleated cells are presented in a median-quartile boxplot; *P < 0.05

Mononuclear cell fusion is a critical step in OC development, and defective preOC fusion stimulates osteopetrosis in mice^24^. Following the exclusion of defective migration in *Ninj1*^−/−^ cells, which could affect the fusion capacity of myeloid cells, we suspected that Ninj1 deficiency interrupted cell–cell fusion itself. Accordingly, we cultured BMMs with M-CSF + IL-4 or GM-CSF + IL-4 for 6 days^25^. Multinucleated giant cells and their nuclei were slightly increased in *Ninj1*^−/−^ cells cultured with M-CSF + IL-4, whereas in GM-CSF + IL-4 culture, 1.44-fold more multinucleated giant cells and significant nuclei increases were observed (Fig. 4). Because Ninj1 deficiency might affect the efficacy of IL-4 in macrophages, we assessed Arginase-1 induction by IL-4 in BMMs; however, no between-group differences were observed (Supplementary Fig. S5a). In addition, preOC migration and/or fusion-associated genes were significantly increased during osteoclastogenesis (Supplementary Fig. S5b). As accelerated myeloid cell fusion reduces bone mass by improving osteoclastogenesis^26,27^, these results suggest that enhanced *Ninj1*^−/−^ cell fusion might counteract osteopetrotic severity in *Ninj1*^−/−^ mice and that Ninj1 has an inhibitory role in myeloid cell fusion.

### Ninj1 deletion reduces mature OC area and attenuates TRAP activity in culture medium

**Fig. 5:**
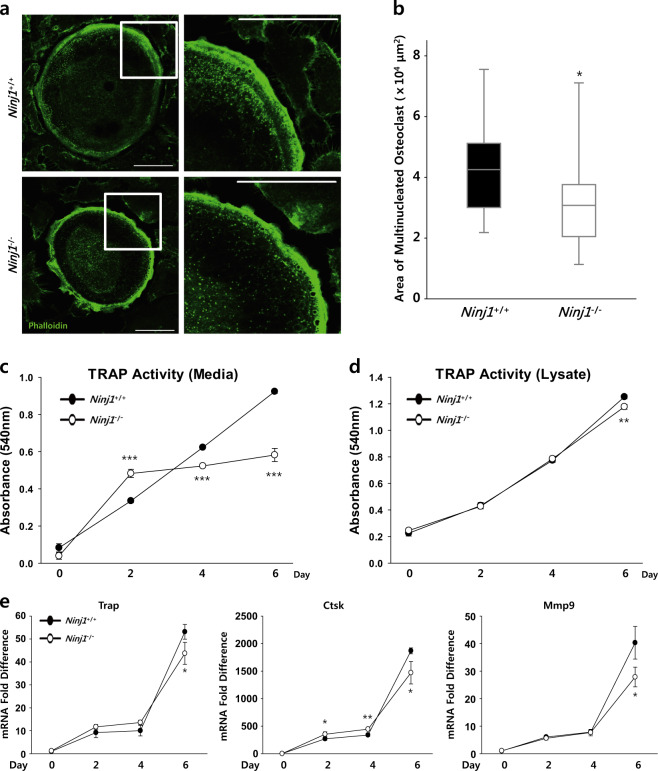
Ninj1-deficient osteoclasts have a smaller area surrounded by an actin ring, and TRAP activity is reduced in *Ninj1*^−/−^ cell culture medium. **a**, **b** BMMs were cultured on glass coverslips with M-CSF and RANKL for 6 days. Cells were stained with AF488-Phalloidin. **a** Representative images of osteoclasts with actin ring formation (left) and highly magnified images of the insets on the left (right). Scale bars, 50 μm. **b** The area inside the actin ring formed in osteoclasts is shown in a median-quartile boxplot; *P < 0.05. **c**, **d** BMMs were cultured with M-CSF and RANKL for the indicated days. Absorbance at 540 nm was measured to assess TRAP activity. **c** Conditioned medium was collected on the indicated days and subjected to a TRAP activity assay. The absorbance is shown as the mean ± SD (*n* *=* 3, with triplicates in each experiment); ***P < 0.001. **d** Cell lysates were harvested on the indicated days and subjected to a TRAP activity assay. The absorbance is shown as the mean ± SD (*n* *=* 3, with triplicates in each experiment); **P < 0.01. **e** mRNA expression of resorption-related osteoclast markers, including TRAP (*Trap*), Cathepsin K (*Ctsk*) and MMP-9 (*Mmp9*), was analyzed by quantitative RT-PCR. The relative mRNA expression is shown as the mean ± SD; *P < 0.05, and **P < 0.01

The OC actin ring cytoskeleton isolates the degradative microenvironment from the general extracellular space; failure of actin ring formation contributes to osteopetrosis development^28^. Thus, we assessed actin cytoskeletons in mature OCs. BMMs were cultured with M-CSF and RANKL on glass coverslips. After 6 days, the cells were stained with phalloidin to visualize F-actin. However, actin ring appearance was indistinguishable between *Ninj1*^−/−^ and WT OCs, with similar proportions of actin ring-forming cells (Fig. 5a). Conversely, mature *Ninj1*^−/−^ OCs were smaller than WT OCs, exhibiting a 0.73-fold smaller area surrounded by the actin ring (Fig. 5b). As *Ninj1*^−/−^ myeloid cells showed no defect in cell–cell fusion or actin ring formation, these data suggest that the area reduction in *Ninj1*^−/−^ OCs might be associated with a diminished preOC cell population.

Bone matrix resorption is a novel OC function dependent upon degradative enzyme quantity and mobilization^29^. Specifically, TRAP directly reflects OC resorptive function^30^. Thus, we assessed TRAP activity during OC development by culturing BMMs with M-CSF and RANKL^31,32^. Conditioned medium TRAP activities were significantly reduced in *Ninj1*^−/−^ culture (Fig. 5c), whereas cell lysate activities were comparable to WT (Fig. 5d). In addition, similar TRAP and other OC enzyme mRNA expression patterns, including those of Cathepsin K and MMP-9, were found (Fig. 5e). This discordance in TRAP activity in *Ninj1*^−/−^ cell culture medium reflects reduced numbers of TRAP-producing cells. Together, these data suggest that *Ninj1*^−/−^ cells dropped out prior to OC maturation and that Ninj1 might be important for cell population maintenance.

### Ninj1 enhances preOC survival during osteoclastogenesis

**Fig. 6:**
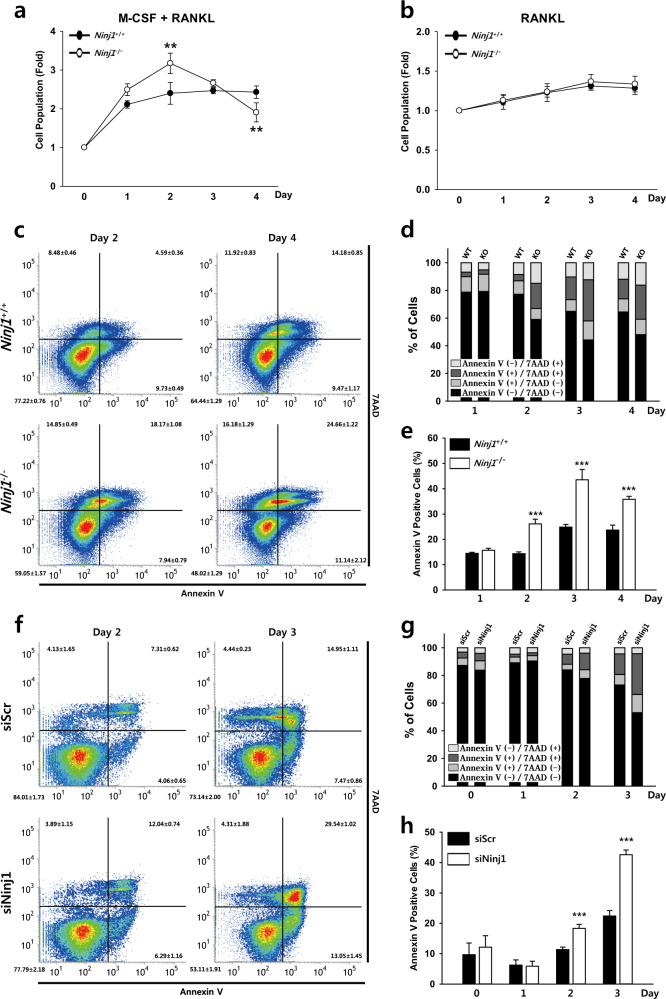
Ninj1 deficiency induces apoptosis during osteoclastogenesis. **a** BMMs were cultured with M-CSF and RANKL. The absorbance induced by MTS was measured at 492 nm, and the relative cell population is shown as the mean ± SD (*n* *=* 3, with triplicates in each experiment); **P < 0.01. **b** BMMs were cultured with RANKL, and the relative cell growth was evaluated at the indicated time points and is shown as the mean ± SD (*n* *=* 3, with triplicates in each experiment). **c**–**e** BMMs were cultured with M-CSF and RANKL. Cells were harvested and stained with FITC-Annexin V and 7AAD on the indicated days followed by FACS analysis. **c** Representative density plots at days 2 and 4. (**d**) FACS-determined statistical stacked bars (*n* *=* 4). (**e**) The FACS-determined percent frequencies of Annexin V^+^ cells are shown as the mean ± SD (*n* *=* 4). (f–h) RAW264.7 cells were transfected with siScr or siNinj1 and cultured with RANKL. Cells were harvested and stained with FITC-Annexin V and 7AAD on the indicated days followed by FACS analysis. (**f**) Representative density plots at days 2 and 3 determined by FACS analysis. (**g**) FACS-determined statistical stacked bars (*n* *=* 4). (**h**) FACS-determined percent frequency of Annexin V^+^ cells. The data are shown as the mean ± SD (*n* *=* 4); ***P < 0.001

Collectively, the previous data suggest that Ninj1 deficiency might influence the cell population by modulating proliferation and/or survival. OC number is influenced by precursor cell propagation and precursor, prefusion, and mature OC death. Thus, we monitored the cell population following M-CSF and RANKL treatment singly or in combination. *Ninj1*^−/−^ BMMs expanded more in culture with M-CSF (Supplementary Fig. S6a) and grew faster during the first 2 days than WT cells with combination treatment. However, from day 2 to day 4, *Ninj1*^−/−^ cells diminished rapidly (Fig. 6a). Neither phenomenon was observed upon RANKL treatment (Fig. 6b). These data indicate that M-CSF-dependent *Ninj1*^−/−^ myeloid cell amplification might ameliorate mature OC reduction *in vitro* and increase trabecular bone volume *in vivo* and that the *Ninj1*^−/−^ cell reduction is related to osteoclastogenic conditions.

To determine whether the population diminishment is related to apoptosis, cells stained with Annexin V and 7AAD were analyzed by FACS. *Ninj1*^−/−^ cell Annexin V^+^ populations were 1.82-, 1.75-, and 1.51-fold greater than those of WT cells at days 2, 3, and 4, respectively; over half of Annexin V^+^ cells were also 7AAD^+^, indicating irreversible apoptosis (Fig. 6c–e). Similar results were obtained in RAW264.7 cells at days 2 and 3 (1.61- and 1.90-fold increase, respectively) following Ninj1 siRNA treatment (Fig. 6f–h). Furthermore, RANKL but not TNF-α specifically increased apoptosis in Ninj1-deficient cells (Supplementary Fig. S6b–e). These results indicate that Ninj1 sustains preOC survival and inhibits apoptosis following OC differentiation.

### Ninj1 deficiency induces Caspase-9-dependent intrinsic apoptosis in preOCs

**Fig. 7:**
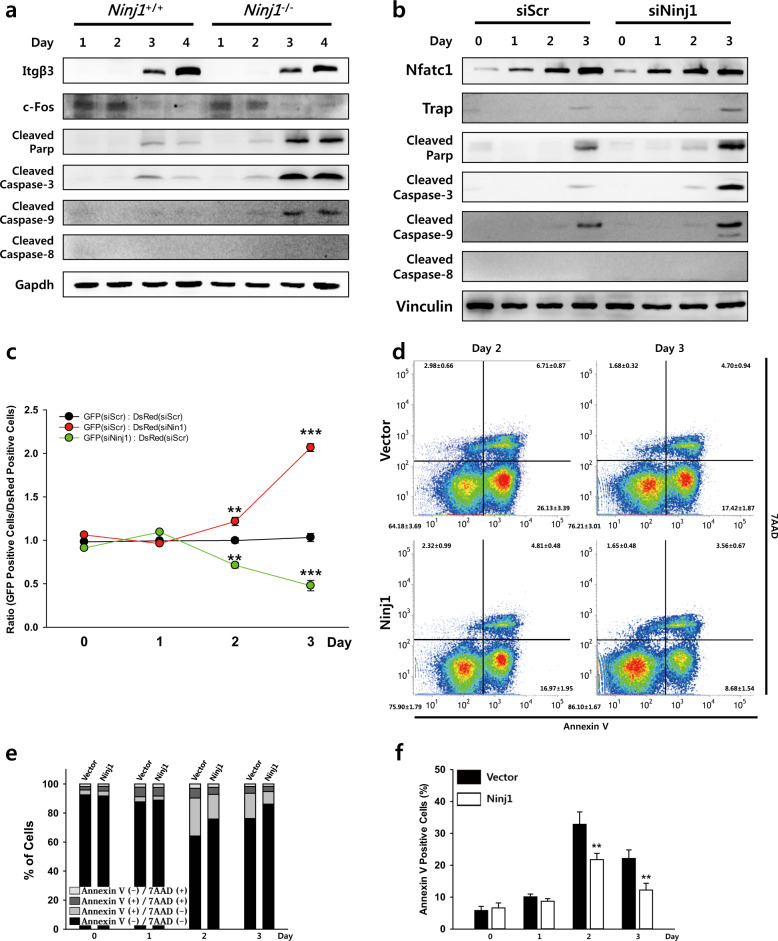
Ninj1 suppresses Caspase-9-dependent intrinsic apoptosis in preOCs, and incremental Ninj1 expression enhances the survival of preOCs. **a** BMMs were cultured with M-CSF and RANKL for the indicated days. Cell lysates were subjected to immunoblot analysis. **b** RAW264.7 cells transfected with siScr or siNinj1 were cultured with RANKL. Cells were harvested on the indicated days and subjected to immunoblot assays. **c** GFP^+^ or DsRed^+^ RAW264.7 cells were transfected with scrambled siRNA or Ninj1 siRNA as indicated. An equal number of each fluorescence protein-labeled cell population was cocultured with RANKL. The ratio of GFP^+^ to DsRed^+^ cells was determined using FACS, and the data are shown as the mean ± SD (*n* *=* 3, with triplicates in each experiment); **P < 0.01, and ***P < 0.001. **d**–**f** RAW264.7 cells were stably transduced with Ninj1-FLAG or empty vector using a retrovirus and cultured with RANKL. Cells were harvested and stained with APC-Annexin V and 7AAD on the indicated days followed by FACS analysis. **d** Representative plots showing the results of FACS analysis on days 2 and 3. **e** FACS-determined statistical stacked bars (*n* *=* 4). **f** FACS-determined percent frequency of Annexin V^+^ cells. The data are shown as the mean ± SD (*n* *=* 4); **P < 0.01

The intrinsic and extrinsic apoptosis pathways are initiated by pro- or antiapoptotic protein dysregulation or liganded death receptors, respectively, followed by sequential activation of Caspase-9 and Caspase-3 or Caspase-8 and Caspase-3^33,34^. To identify the type of apoptosis induced by Ninj1 deficiency, cell lysates from BMMs cultured with M-CSF and RANKL were immunoblotted. Cleaved Caspase-3 was observed from day 2 in *Ninj1*^−/−^ cells and was augmented following differentiation. Notably, cleaved Caspase-9 but not cleaved Caspase-8 was observed with cleaved Caspase-3 and cleaved PARP in *Ninj1*^−/−^ cells (Fig. 7a). Similarly, Caspase-9 and Caspase-3 active forms were stronger in siNinj1-transfected vs. control RAW264.7 cells without cleaved Caspase-8 detection (Fig. 7b). To determine whether Ninj1-deficient cell apoptosis might affect neighboring cell viability or was cell-autonomous, GFP- or DsRed-labeled RAW264.7 cells were transfected with siScr or siNinj1 and an equal number of GFP^+^ or DsRed^+^ transfectants were cultured together with RANKL. The ratio of GFP^+^ to DsRed^+^ cells indicated that neither siNinj1 nor control transfectants impacted the respective viability of the other (Fig. 7c, Supplementary Fig. S7a,b). To ascertain whether an increase in Ninj1 expression would attenuate spontaneous apoptosis during osteoclastogenesis, we transduced RAW264.7 cells with Ninj1-FLAG and following RANKL treatment examined apoptosis via FACS. Annexin V^+^ cells were 0.66- and 0.55-fold reduced by Ninj1 overexpression at days 2 and 3, respectively (Fig. 7d–f, Supplementary Fig. S7c). Collectively, these data indicate that Ninj1 is a prerequisite for preventing Caspase-9-dependent apoptosis induction in preOCs and that Ninj1 upregulation enhances preOC survival.

### High *NINJ1* expression is correlated with human bone disorders

**Fig. 8:**
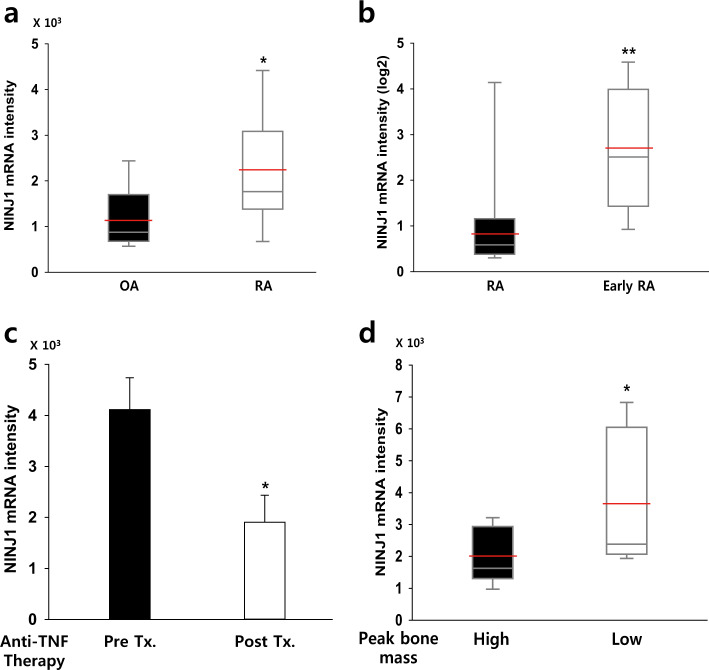
The expression level of NINJ1 is correlated with bone disorders in humans. **a**–**d** Expression levels of *NINJ1* mRNA were analyzed from the gene expression data sets GSE27390 (**a**), GSE1964 (**b**), GSE7524 (**c**) and GSE7158 (**d**) deposited in GEO. Red bars indicate the mean value. **a** Bone marrow-derived mononuclear cells were obtained from patients with osteoarthritis (*n* = 10) or rheumatoid arthritis (*n* = 9), and *NINJ1* mRNA expression was analyzed. The data are shown in a median-quartile boxplot; *P < 0.05. **b** Comparison of *NINJ1* expression in peripheral blood mononuclear cells derived from patients with rheumatoid arthritis (*n* *=* 8) or early rheumatoid arthritis (*n* = 15). The data are shown in a median-quartile boxplot; **P < 0.01. **c** The change in *NINJ1* mRNA expression in whole blood samples from rheumatoid arthritis patients following anti-TNF therapy was analyzed, and the data are shown as the mean ± SD (*n* *=* 2); *P < 0.05. **d** The difference in *NINJ1* mRNA expression in circulating monocytes derived from postmenopausal women with high (*n* *=* 8) and low (*n* *=* 7) peak bone mass was analyzed, and the data are shown in a median-quartile boxplot; *P < 0.05

To investigate the potential association between *NINJ1* expression and bone disorders in humans, we mined publicly available data sets in GEO and analyzed four different microarrays. Gene expression profiles of 10 patients with osteoarthritis (OA) and 9 with rheumatoid arthritis (RA) demonstrated significantly elevated *NINJ1* expression in RA (1.99-fold compared with OA, Fig. 8a). Furthermore, comparison of *NINJ1* expression in 8 patients with RA and 15 with early RA revealed a 2.60-fold enhancement in early RA, suggesting that *NINJ1* is important for RA onset (Fig. 8b). Following three months of anti-TNF (Enbrel) therapy in 2 patients with RA, *NINJ1* expression was significantly reduced (0.46-fold vs. pretreatment, Fig. 8c). Furthermore, postmenopausal women with low peak bone mass (*n* *=* 7) exhibited 1.40-fold higher *NINJ1* expression than those with high peak bone mass (*n* *=* 8), suggesting that *NINJ1* might be associated with osteoporotic bone loss (Fig. 8d). Together, these data suggest that *NINJ1* has a potent role in human bone disorder pathogenesis and/or progression.

## Discussion

The current study investigated the role of Ninj1 in OC development and cell survival maintenance, providing the first evidence that Ninj1 is important for bone homeostasis by sustaining preOC survival. We show that Ninj1 is highly expressed in OCs differentiated from bone marrow and that OC and macrophage Ninj1 expression is comparable. Plain X-ray images demonstrated strong hind limb intensity and linear rather than concave femur diaphysis morphology in *Ninj1*^−/−^ mice, which are novel signs of osteopetrosis^35^. μCT analysis further demonstrated increased femoral and tibial trabecular bone mass in *Ninj1*^−/−^ mice, which was prolonged from 6- to 12-weeks. *Ninj1*^−/−^ mice have been reported to be small and have several skeletal deformities, such as an asymmetric face or dome-shaped head, and often develop hydrocephalus^14,36^, which is frequently developed in patients with autosomal recessive osteopetrosis^8,37^. In addition, *Ninj1*^−/−^ mice frequently exhibit vertebral column (kyphosis or scoliosis) and orbital bone (cyclopia) deformities and often show cycling behavior, suggesting the possibility of defective ossicles and/or cochlea (unpublished observations). Collectively, these data suggest that Ninj1 might impact bone homeostasis by modulating OC development and/or function.

OCs accomplish bone resorption through several continuous processes: (1) OC progenitor/precursor cell development from hematopoietic stem cells, (2) OC precursor proliferation, (3) OC precursor differentiation into mononuclear preOCs, (4) preOC fusion into multinucleated OCs, (5) attachment to calcified matrix and OC polarization followed by matrix degradation, and finally (6) death by apoptosis^38^. Our data show that Ninj1 deficiency reduces the number of TRAP^+^ multinucleated OCs derived from bone marrow cells but does not impact differentiation into OCs as defined by OC marker expression, suggesting that Ninj1 deficiency only minimally disrupts major OC differentiation signaling pathways, such as MAPK, AP-1, NFκB, and NFATc1^39^. Therefore, the reduction in mature OCs might result from a decreased proportion or quality of OC progenitor cells in the bone marrow, which are derived from hematopoietic stem cells that give rise to common lymphoid and myeloid progenitors in response to various factors^40^. Accordingly, M-CSF, PU.1, MITF, and Bcl-2 mutation lead to osteopetrosis in mice resulting from reduced OC progenitor cells^41^. Specifically, c-Kit^+^c-Fms^+^CD11b^dull^ cells are potent bone marrow OC progenitor cells that effectively differentiate into OCs^19^. However, c-Kit^+^c-Fms^+^CD11b^dull^ OC progenitor cells are clearly increased in *Ninj1*^−/−^ mice. Nonetheless, whereas we previously showed similar peripheral blood cell composition in *Ninj1*^−/−^ and WT mice via complete blood counts, the differential count identified a trend toward myeloid cell increase, including neutrophils (1.28-fold) and monocytes (1.33-fold)^14^. Combined with the current observations, these data suggest that Ninj1 might instead have a potent role in myelopoiesis. Finally, the essential osteoclastogenic cytokines M-CSF and RANKL bind M-CSF receptor and RANK, respectively, on OC precursors, and their targeted deletion leads to osteopetrosis in mice caused by OC differentiation failure^42–44^. However, our data show that Csf1r, Rank, and Cd11b expression in BMMs is indistinguishable between genotypes, indicating that *Ninj1*^−/−^ OC precursor cells develop normally from myeloid progenitors and implying that Ninj1 deficiency might affect a later stage of osteoclastogenesis.

OC precursors exposed to M-CSF and RANKL differentiate into mononuclear preOCs followed by mononuclear cell fusion to generate multinucleated osteoclasts^6^. Disruption of cell fusion through genetic ablation of ATP6v0d2^45^ or DC-STAMP^46^, which are suspected to be involved in cell–cell recognition^47^, results in severe osteopetrosis in mice due to multinucleated osteoclast deficiency. As Ninj1 is a homophilic adhesion protein^12^, Ninj1 deficiency might also disrupt cell–cell recognition and fusion. However, *Ninj1*^−/−^ BMMs fuse more efficiently than WT BMMs, especially in culture with GM-CSF + IL-4. Furthermore, the expression of several motility- and fusion-associated genes was enhanced in *Ninj1*^−/−^ cells, and transmigration capabilities were indistinguishable between genotypes, suggesting that Ninj1 is dispensable for preOC migration and fusion. Additionally, the frequency of multinucleated cells with an actin ring cytoskeleton and the actin ring morphologies are indistinguishable between WT and *Ninj1*^−/−^ cells. Conversely, the area enclosed by the actin ring is significantly reduced in *Ninj1*^−/−^ OCs, suggesting that the participant mononuclear preOC numbers might be decreased by Ninj1 deficiency prior to this stage. The significant reduction in TRAP activity in the *Ninj1*^−/−^ cell conditioned medium but not in cell lysate indicates that Ninj1 deletion might alter the cell population by modulating proliferation and/or survival.

Aberrant OC apoptosis leads to bone homeostasis dysregulation and bone disease. OC apoptosis is lower in osteoporotic bone and evident in the late stage of Paget’s bone disease, which manifests as sclerotic bone lesions^48^. Our data revealed that Ninj1 deficiency induces Caspase-9-dependent intrinsic apoptosis in both primary and RAW264.7 cells in a cell-autonomous manner. Ninj1 reduction accompanied apoptotic features in WT preOCs, and Ninj1 overexpression in RAW264.7 cells reduced this spontaneous apoptosis in osteoclastogenic culture. Thus, during osteoclastogenesis, the Ninj1 level influences preOC survival, with deficiency exacerbating apoptosis. Conversely, loss of Ninj1 in BMMs accelerates M-CSF-induced cell proliferation. The novel role of Ninj1 in OC/macrophage precursor cell propagation needs to be elucidated.

Recently, a protective role of Ninj1 against different types of cell death was reported. Ninj1 inhibited p53-dependent cellular senescence by reducing p53 translation, potentially regulated by mechanistic targeting of rapamycin (mTOR) kinase^36,49^. M-CSF and RANKL promoted OC survival through mTOR kinase by downregulating the expression of Bim, a downstream effector of PI3K^50,51^. Conversely, p53 inhibited mTOR, resulting in suppression of cell survival^52,53^. Furthermore, *p53*^−/−^ mice exhibit increased OC number and bone resorption^54^. Thus, Ninj1 might potentially enhance preOC survival by intervening in p53-mTOR kinase crosstalk. Considering its role as a c-Jun-regulated antiapoptotic gene, apoptosis was induced by Ninj1 downregulation in triple-negative breast cancer cells and further accelerated by TNF-α, whereas Ninj1 overexpression ameliorated TNF-α-induced apoptosis in c-Jun knockdown cells^55^. However, in our study, TNF-α did not induce apoptosis in Ninj1-deficient RAW264.7 cells; this discrepancy might result from different TNF receptor occupancy in immune cells. Nevertheless, our data support the protective role of Ninj1 against various types of cell death.

Decreased OC apoptosis is implicated in destructive bone diseases, such as RA and osteoporosis^56^. In bone destructive disorders, TNF-α is commonly elevated and participates in progressive bone resorption^57^. Increased TNF-α enhances the survival and differentiation of osteoclasts^50^, which exacerbates bone loss, and blockade of TNF-α provides a powerful therapeutic strategy for bone destructive disorders^58,59^. Our data show that not only RANKL but also TNF-α upregulated Ninj1 expression in BMM. In addition, increased Ninj1 expression ameliorated spontaneous apoptosis during osteoclastogenesis in RAW264.7 cells, and microarray analyses revealed correlations between high *NINJ1* expression and the above disorders.

Moreover, an increase in Ninj1 might play a detrimental role in destructive bone disorders as well as in other pathologic conditions, such as diabetes mellitus^60^. Accordingly, *NINJ1* is associated with insulin insensitivity and type 2 diabetes incidence in African Americans^61^. In humans, diabetes elicits several metabolic and endocrine alterations that result in osteoporosis^62^, and streptozotocin induces osteoporosis following the occurrence of diabetes in mice^63^. Therefore, an investigation into the precise role of NINJ1 in bone homeostasis in metabolic diseases such as diabetes might yield new insights into the systemic regulation of bone metabolism.

The hallmark of OCs is their unique ability to resorb mineralized calcium apatite or carbonate substrates in bone^7^. However, in the current study, we primarily focused on the role of Ninj1 in OC development. In our mice, Ninj1 is ubiquitously deleted so that during *in vitro* OC differentiation, most *Ninj1*^−/−^ cells dropped out and a few mature OCs remained. The failure to provide mature OCs rendered defining the role of Ninj1 in those cells difficult. Accordingly, stage-specific Ninj1 deletion might extend the current findings to mature OCs.

This work presents several novel findings. First, Ninj1 is highly expressed in OCs in contrast with OBs, and *Ninj1*^−/−^ mice show a mild osteopetrotic phenotype in long bones. Second, Ninj1 deficiency reduces multinucleated OC numbers. Third, *Ninj1*^−/−^ preOCs are vulnerable to Caspase-9-dependent intrinsic apoptosis. Finally, high *NINJ1* expression is correlated with human bone disorders, such as RA and postmenopausal osteoporosis. These findings have illuminated a novel function of Ninj1 in bone homeostasis, suggesting that Ninj1 is one of the several molecular regulators of osteoclast development and thus represents a potential therapeutic target in human destructive bone disorders.

## Supplementary information

Supplementary Information
