# Author Correction: Ninjurin1 positively regulates osteoclast development by enhancing the survival of prefusion osteoclasts

**DOI:** 10.1038/s12276-025-01475-4

**Published:** 2025-06-04

**Authors:** Sung-Jin Bae, Min Wook Shin, Taekwon Son, Hye Shin Lee, Ji Soo Chae, Sejin Jeon, Goo Taeg Oh, Kyu-Won Kim

**Affiliations:** 1 College of Pharmacy and Research Institute of Pharmaceutical Sciences, Seoul National University, Seoul, 08826 Korea; 2 Korean Medicine Research Center for Healthy Aging, Pusan National University, Yangsan, 50612 Korea; 3 RNA Therapeutics Institute, University of Massachusetts Medical School, Worcester, MA USA; 4 Department of Life Sciences and Technology, PerkinElmer, Seoul, 06702 Korea; 5 Department of Life Sciences, Ewha Womans University, Seoul, 03760 Korea; 6 Crop Biotechnology Institute, GreenBio Science and Technology, Seoul National University, Pyeongchang, 25354 Korea

Correction to: *Experimental & Molecular Medicine* (2019) **51**(1), 7; 10.1038/s12276-018-0201-3, published online 16 January 2019

After online publication of this article, the authors noticed an error in the Fig. 3d section.

In Fig. 3d, the authors identified that the WT result (12 hr, lower left) was unintentionally duplicated with the *Ninj1*^−/−^ result (12 hr, upper right) during the data organization process. To rectify this duplication, we prepared the correct version of this figure with the proper *Ninj1*^−/−^ result (12 hr, upper right). Fig. 3d reveals that transmigration of RANKL-stimulated preosteoclast (red) through the osteoblast monolayer (green) is similar between WT and Ninj1 KO. Thus, our conclusion of a crucial role of Ninj1 in preosteoclast survival is not influenced by the correction.

The authors apologize for any inconvenience caused.

**Fig. 3d in the article**. Duplicated images were marked with red rectangles.
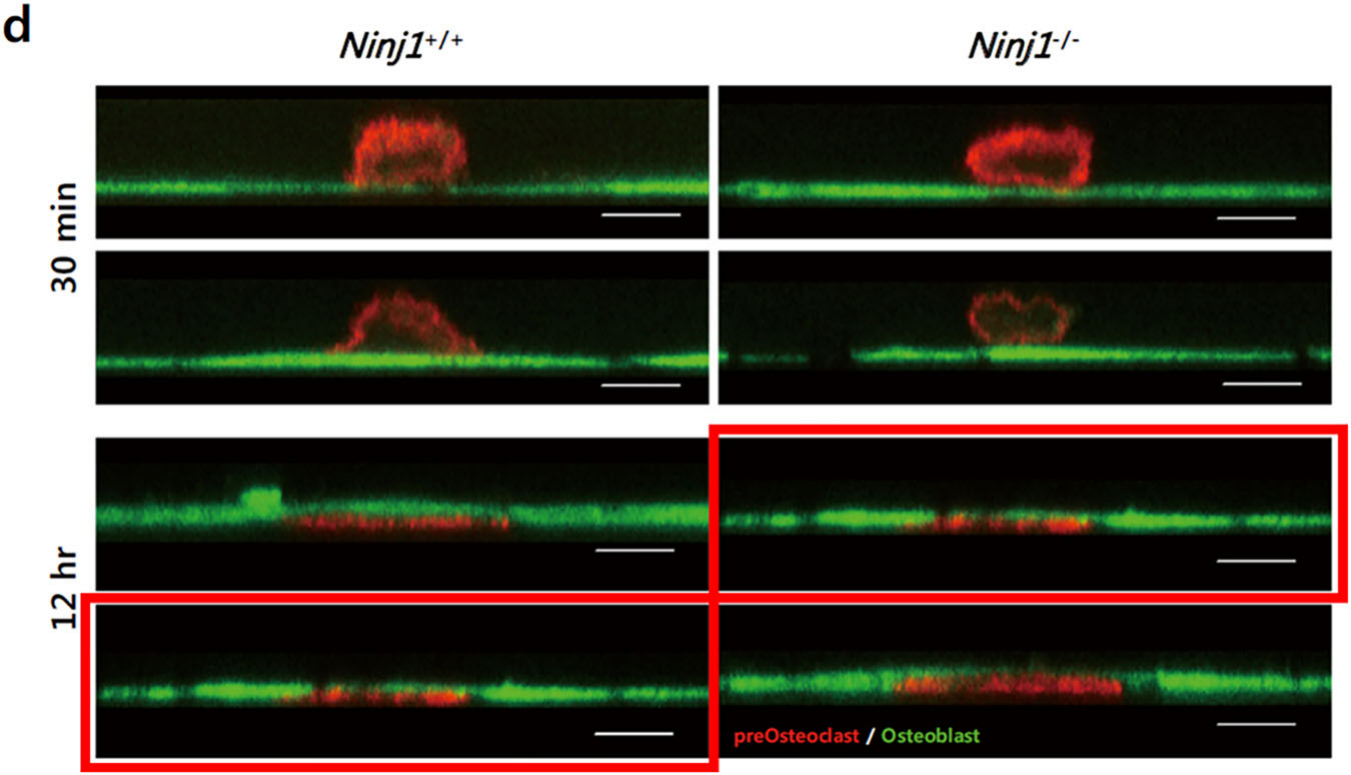


**Corrected Fig. 3d**. The proper result of *Ninj1*^−/−^ (12 hr, upper right) was included.
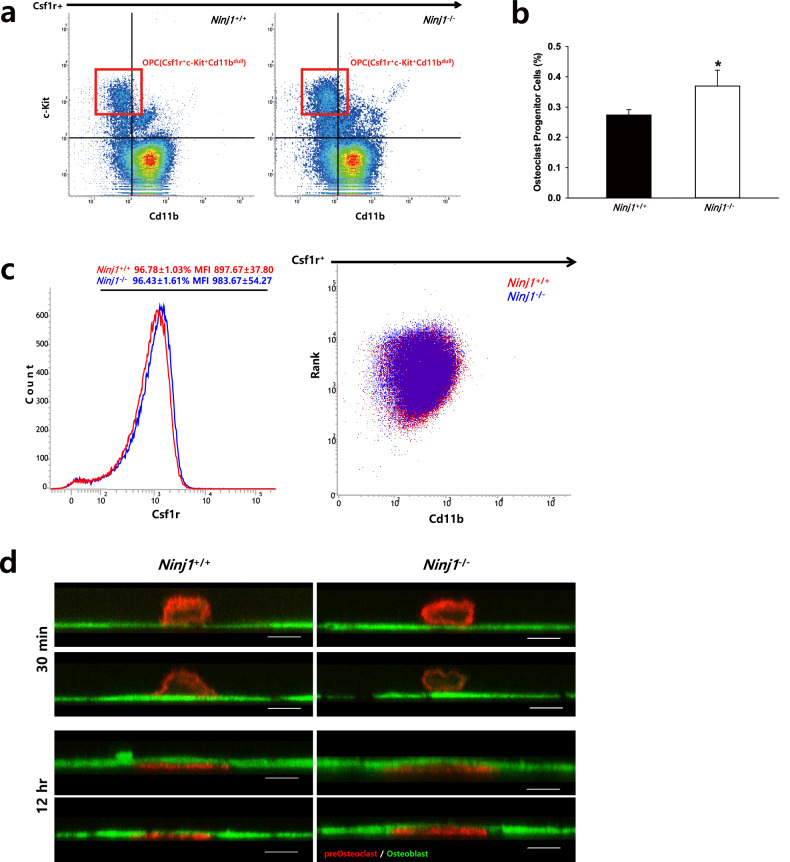


The original article has been corrected.

