# Supplementary material for: Ninjurin1 positively regulates osteoclast development by enhancing the survival of prefusion osteoclasts
Source: Exp Mol Med. 2019 Jan 16;51(1):1–16. doi: 10.1038/s12276-018-0201-3 (PMC6353902; doi:10.1038/s12276-018-0201-3)
Supplement: Supplementary file 1 — Supplementary Information [file 12276_2018_201_MOESM1_ESM.pdf]

Supplementary information

Ninjurin1 positively regulates osteoclast development by enhancing the survival of prefusion osteoclasts

Sung-Jin Bae[1], Min Wook Shin[1], Taekwon Son[1], Hye Shin Lee[1], Ji Soo Chae[2], Sejin Jeon[3], Goo Taeg Oh[3], Kyu-Won Kim[1,4,*]

[1]College of Pharmacy and Research Institute of Pharmaceutical Sciences, Seoul National University, Seoul 08826, Korea
[2]Department of Life Sciences and Technology, PerkinElmer, Seoul 06702, Korea
[3]Department of Life Sciences, Ewha Womans University, Seoul 03760, Korea
[4]Crop Biotechnology Institute, GreenBio Science and Technology, Seoul National University, Pyeongchang 25354, Korea

*Correspondence:
Kyu-Won Kim, Ph.D.
College of Pharmacy and Research Institute of Pharmaceutical Sciences, Seoul National University, Seoul 08826, Korea
Phone: 82-2-880-6988
Fax: 82-2-885-1827
E-mail: qwonkim@snu.ac.kr

Supplementary materials and methods

Microcomputed tomography

Tibias isolated from 6- or 12-week-old mice were scanned individually by radiology apparatus. Plain radiologic images of hind limbs were acquired by IVIS Spectrum CT with Living Image Software 4.4.0. (PerkinElmer, Waltham, MA) and μCT images were obtained by Quantum GX microCT (PerkinElmer) at 10-μm isotropic resolution and were processed with Caliper Viewer 1.3.0 (PerkinElmer). Trabecular bone volume was analyzed over 1-mm lengths encompassing the region of proximal metaphysis away from the distal edge of the growth plate in proximal end of tibias using Inveon Research Workplace (Siemens, Malvern, PA).

Plasmid construction, transfection, and retroviral infection

For Ninj1-FLAG, the coding region of mouse Ninj1 (NM_013610.2) was amplified by PCR and cloned into pMX-IRES-GFP vector with C-terminal tagged FLAG sequence. For Lifeact-mCherry, sense and antisense oligonucleotides of Lifeact sequence [1] were annealed and cloned into pMX-IRES-Puromycin^R vector with PCR-amplified mCherry sequence. For GFP and DsRed, each sequence was amplified by PCR and cloned into pMX vector. Retroviral packaging was performed by transfecting retroviral vectors into Platinum A or E cells with linear polyethylenimine (Polysciences, Warrington, PA). Cultured media containing viral particle were collected after 48 hours and filtered through 0.45-μm syringe filters. Subsequently, target cells were cultured in virus containing media with polybrene (5 μg/mL) (Sigma-Aldrich, St. Louis, MO). Transduced cells were selected with puromycin (1 μg/mL) for pMX-Lifeact-mCherry-IRES-Puromycin^R or by FACS sorting for pMX-GFP, pMX-DsRed, pMX-IRES-GFP, and pMX-Ninj1-FLAG-IRES-GFP with FACSAria III (BD Biosciences, Bedford, MA). Sequence of Ninj1 and Scrambled siRNA was described previously (Shin et al., 2016). For knockdown experiments, RAW264.7 cells were transfected with either Ninj1 or Scrambled siRNA using Lipofectamine RNAiMAX (Invitrogen).

Cell culture

MC3T3-E1 cells were maintained in α-MEM supplemented with 10% FBS. For establishing stable GFP expression in MC3T3-E1 cells, the cells were transduced with GFP by retrovirus. GFP or DsRed expressing RAW264.7 cells were established by retroviral transduction. For stable expression of Ninj1, RAW264.7 cells were transduced with retrovirus containing Ninj1-FLAG-IRES-GFP. The expression of Ninj1-FLAG was confirmed by immunoblot assay and Ninj1-overexpressing RAW264.7 cells were conducted to Annexin V and 7AAD analysis. In this case, APC-Annexin V (Biolegend) was applied instead of FITC-Annexin V.

RNA isolation and quantitative RT-PCR

Total RNA was isolated with TRIzol reagent (Invitrogen) and cDNA was obtained from 2 µg of total RNA using MMLV reverse transcriptase (Promega Corporation, Madison, WI). Quantitative RT-PCR was then performed using StepOnePlus RT-PCR system (Applied Biosystems, Foster City, CA) with RealHelix qPCR kit (NanoHelix, Seoul, Korea). Relative mRNA expression levels were calculated by the comparative $2^{-\Delta\Delta Ct}$ method. *Gapdh* and/or *Hprt* served as an internal control. Data are expressed as relative mRNA expression. Primer sequences and conditions for PCR are summarized in Table S1.

Immunoblot assay

Cells were lysed in RIPA buffer containing 20 mM Tris pH 7.5, 150 mM NaCl, 1 mM Na_2EDTA, 1 mM EGTA, 1% NP-40, 1% sodium deoxycholate, phosphatase inhibitor cocktail (Sigma-Aldrich) and proteinase inhibitor cocktail (Calbiochem, San Diego, CA). Protein concentration was determined by BCA assay. Lysates (20 − 40 µg) were resolved on polyacrylamide gel and then immunoblotted as described previously [2].

Proliferation assay

Measure of cell growth by MTS was performed using a Cell Proliferation Assay Kit (Promega) according to manufacturer protocol. In brief, 1×10^4 BMMs/well were seeded on 96-well plates and cultured with M-CSF (30 ng/mL) and RANKL (100 ng/mL) singly or in combination. At the indicated days, 20 µl of substrate solution was added to each well and incubated for 3 hours at 37℃. Reaction was stopped by adding 25 µl of 10% SDS and absorbance at 492 nm was measured using microplate reader (Spectra Fluor; Tecan, San Jose, CA). Data are expressed as fold ratio to days 0.

Supplementary references

1. Riedl J, Crevenna AH, Kessenbrock K, Yu JH, Neukirchen D, Bista M, et al. Lifeact: a versatile marker to visualize F-actin. *Nat Methods* 2008;**5**(7):605-607.

2. Bae SJ, Shin MW, Kim RH, Shin D, Son T, Wee HJ, et al. Ninjurin1 Assembles Into a Homomeric Protein Complex Maintained by N-linked Glycosylation. *J Cell Biochem* 2017;**118**(8):2219-2230.

Figure S1

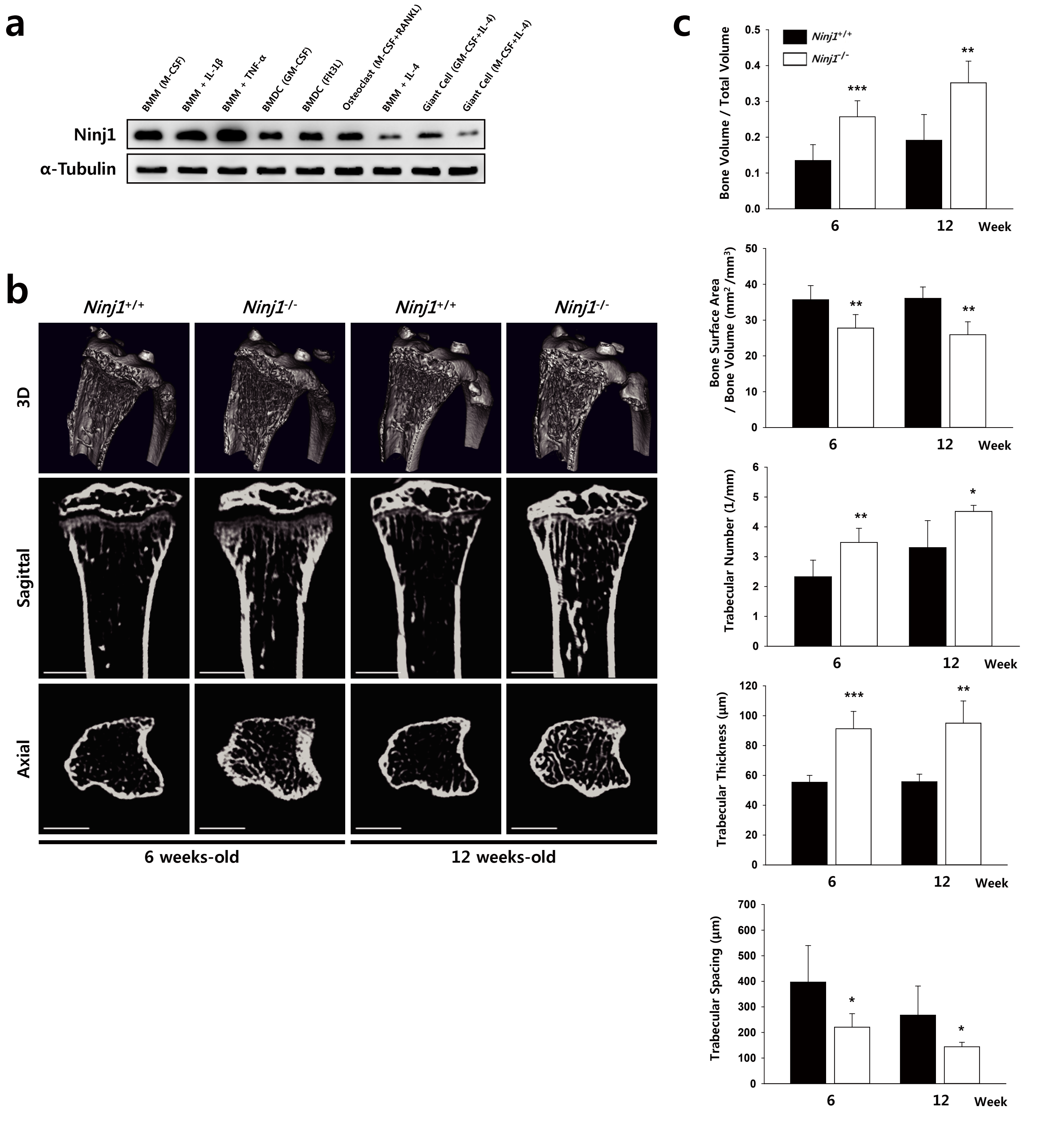

Fig. S1. Ninj1 expression in myeloid cells and comparison of tibias from WT and *Ninj1*$^{-/-}$ mice by microCT. (a) Ninj1 expression was assessed in different myeloid cells by immunoblot assay. (b) Representative μCT images of proximal tibias from *Ninj1*$^{-/-}$ 6- or 12-week-old mice and their corresponding WT littermate mice. Scale bars, 1 mm. (c) μCT-determined trabecular bone parameters of proximal tibias from 6- or 12-week-old WT (n = 5, n = 4, respectively) and *Ninj1*$^{-/-}$ mice (6-weeks-old; n = 7, 12-weeks-old; n = 6). All quantitative data are mean ± SD, *P<0.05, **P<0.01, ***P<0.001.

Figure S2

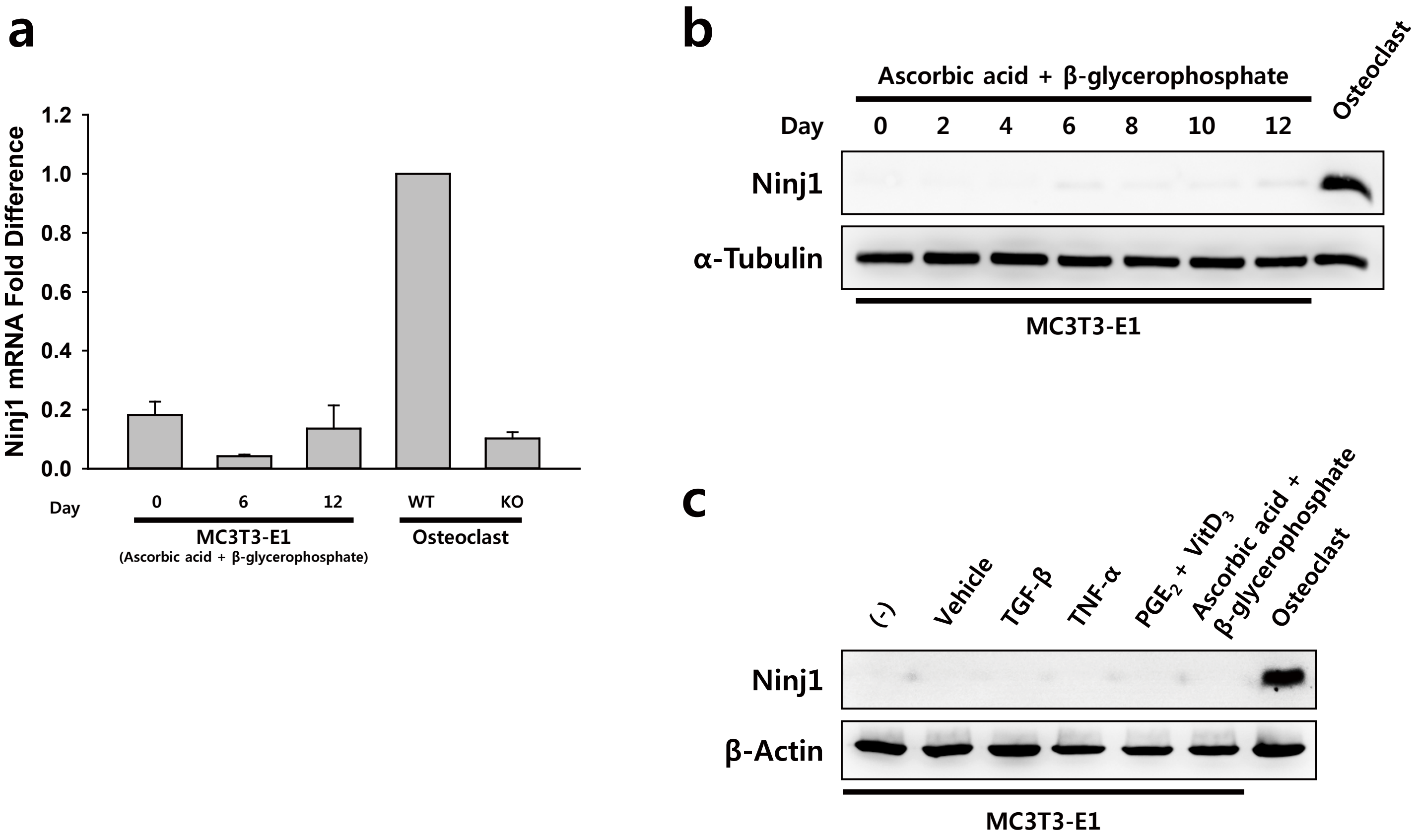

Fig. S2. Analysis of Ninj1 expression in MC3T3-E1 cells. (a) mRNA expression of *Ninj1* in MC3T3-E1 by osteoblastogenic condition. WT and *Ninj1*$^{-/-}$ osteoclasts were used as positive and negative control, respectively. (b) Protein expression of Ninj1 in MC3T3-E1 by osteoblastogenic condition. Lysate from WT osteoclast was used as positive control. (c) Ninj1 expression by variable osteogenic stimuli in MC3T3-E1 was measured by immunoblot assay. Lysate from WT osteoclast was used as positive control.

Figure S3

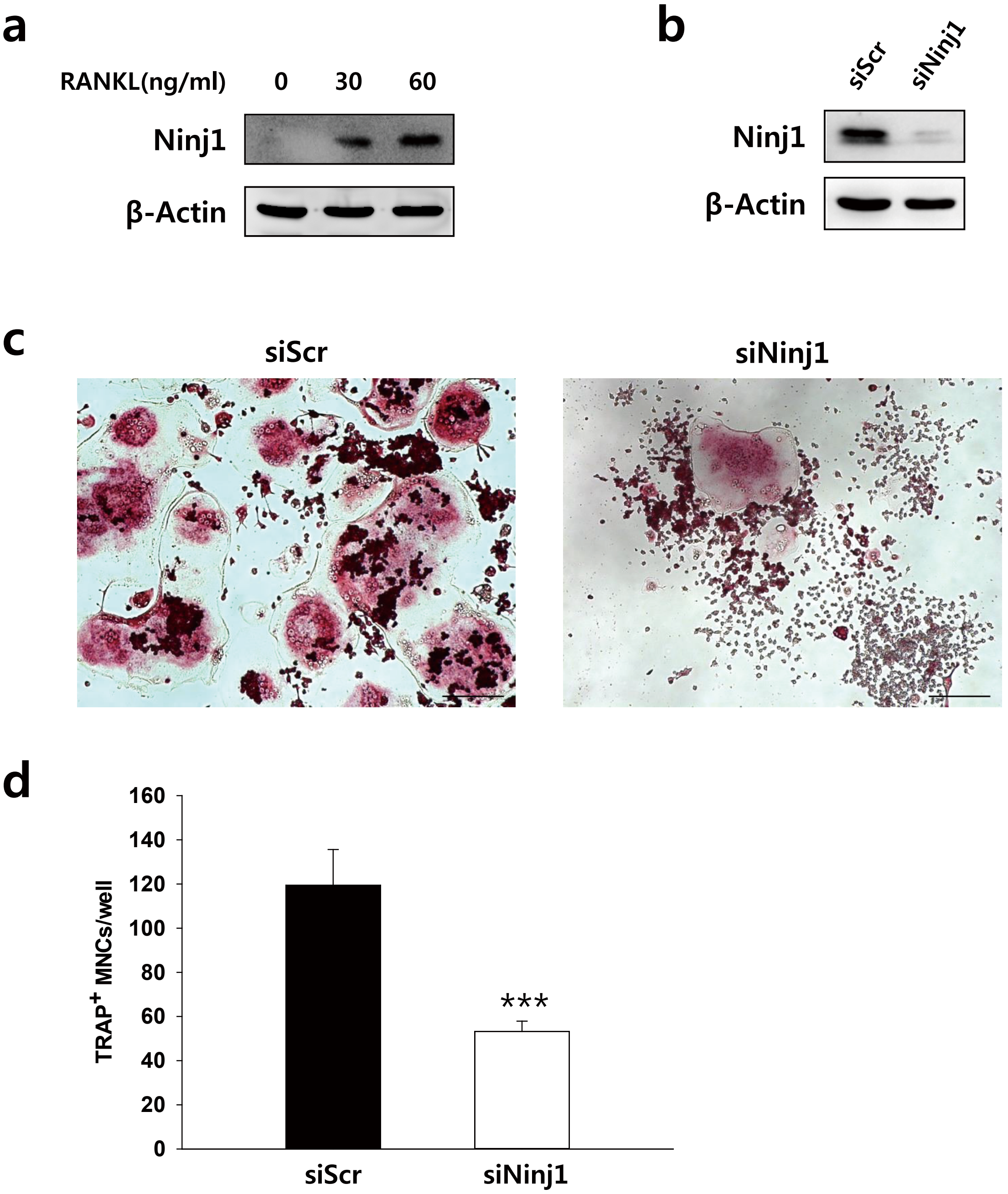

Fig. S3. Ninj1 expression by RANKL and siNinj1-mediated impairment of osteoclast development in RAW264.7 cells. (a) Ninj1 expression by dose-dependent RANKL was evaluated in RAW264.7 cells by immunoblot assay. (b-d) RAW264.7 cells were transfected with siScr or siNinj1 and cultured with RANKL to generate mature osteoclasts. At day 6, the cells were stained for TRAP activity. (b) Downregulation of Ninj1 by siRNA was assessed by immunoblot assay at day 1. (c) Representative images of TRAP activity staining. Scale bars, 200 μm. (d) Quantitation of TRAP-positive multinucleated osteoclasts. Numbers of TRAP-positive multinucleated osteoclasts ($TRAP^+$ MNCs, ≥ 3 nuclei) are shown as mean ± SD, ****P*<0.001.

Figure S4

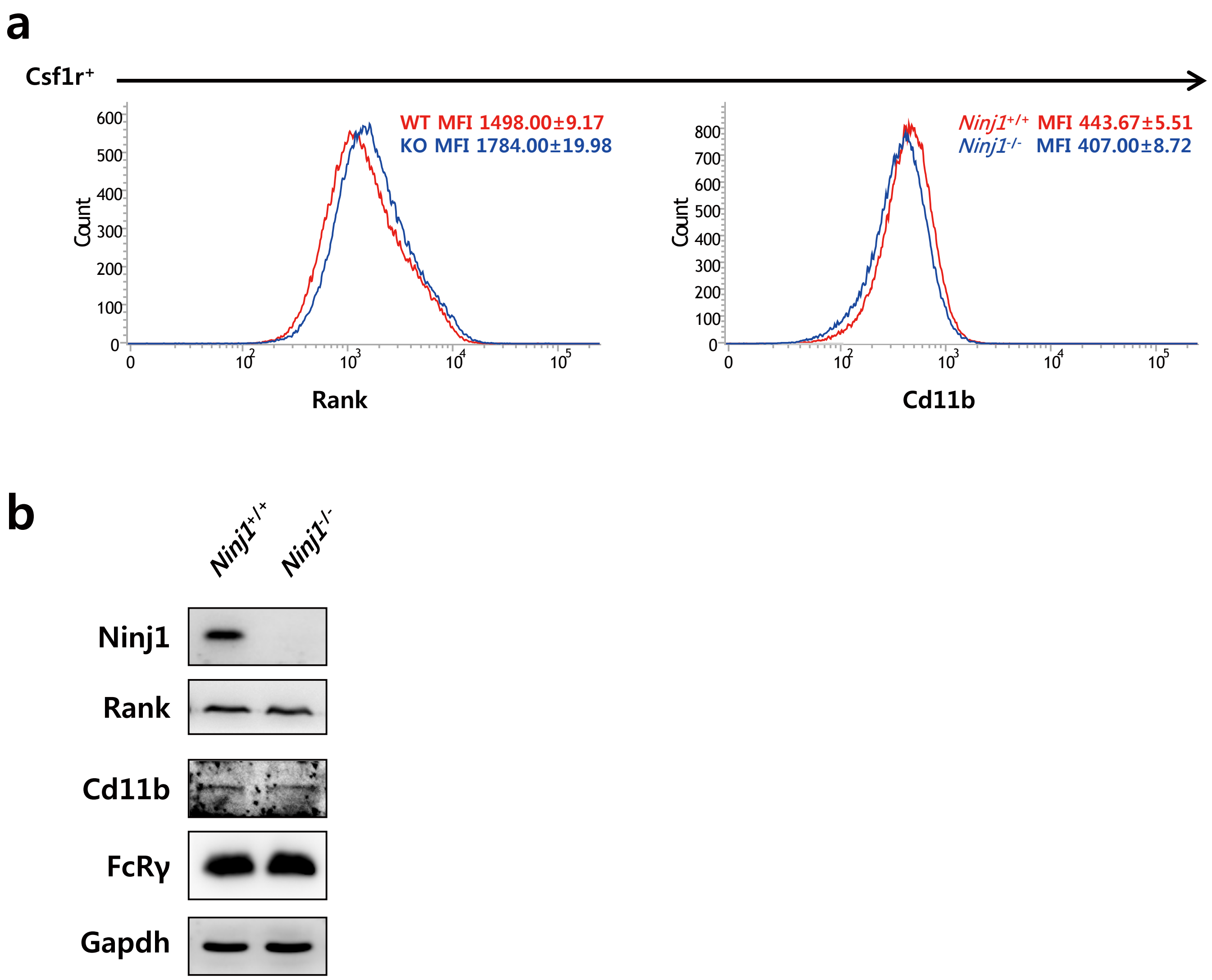

Fig. S4. Expressions of macrophage/osteoclast precursor markers in BMMs. (a) Expressions of Rank (left) or Cd11b (right) were measured in Csf1r^{+} BMMs by FACS. (b) Expressions of Rank, Cd11b, and FcRγ in BMMs were analyzed by immunoblot assay.

Figure S5

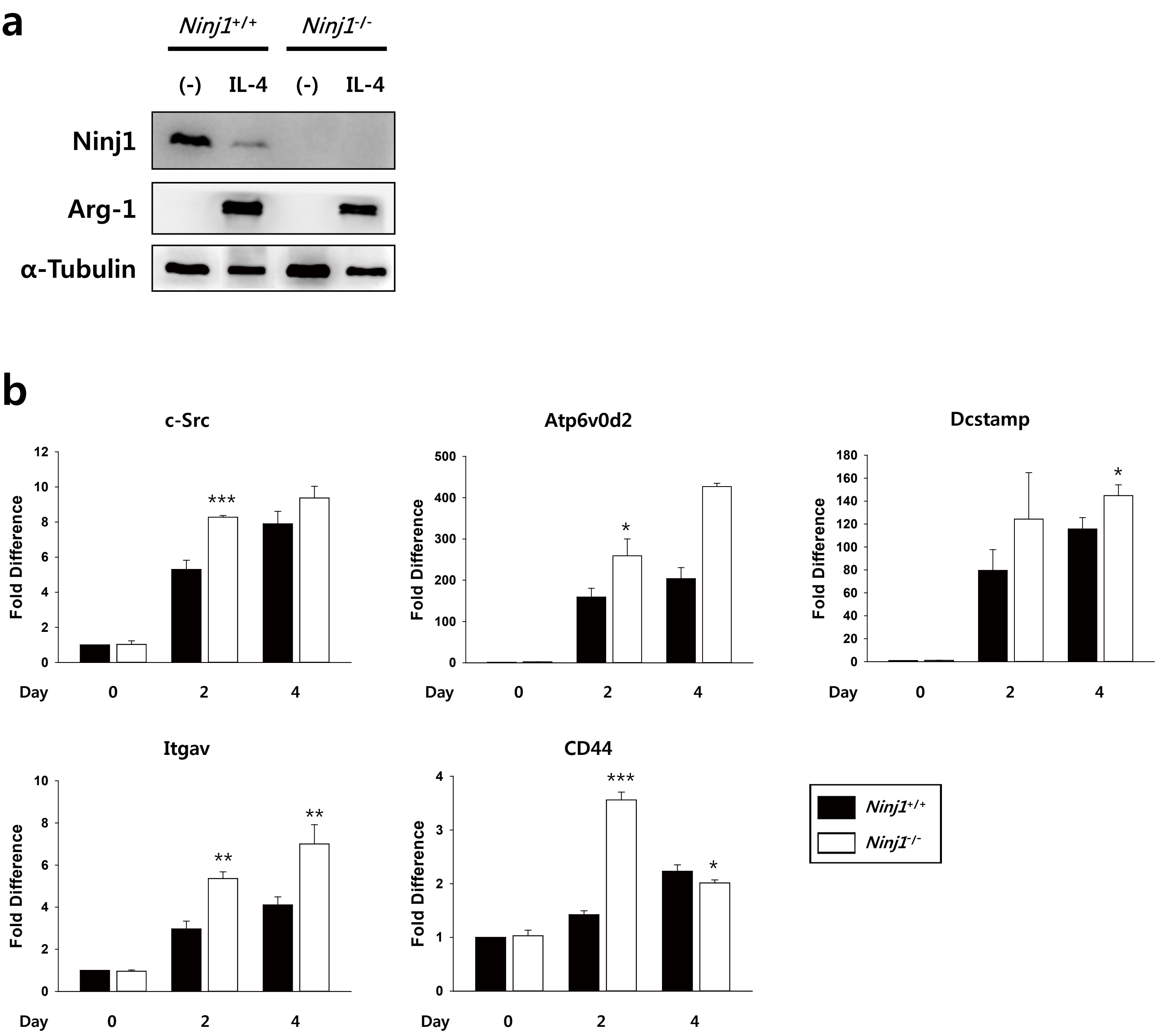

Fig. S5. Arginase-1 expression by IL-4 in BMMs and mRNA expressions of cytoskeleton and/or fusion related genes during osteoclastogenesis. (a) Cytokine-starved BMMs were cultured with or without IL-4 (10 ng/mL) for 24 hours. Expressions of Arg-1 and Ninj1 were evaluated by immunoblot assay. (b) mRNA expressions of cytoskeleton and/or fusion related genes including *c-Src*, *Atp6v0d2* *Dcstamp*, *Itgav*, and *Cd44* in osteoclastogenic culture were analyzed by quantitative RT-PCR. Relative mRNA expressions are shown as mean ± SD, *P<0.05, **P<0.01, ***P<0.001.

Figure S6

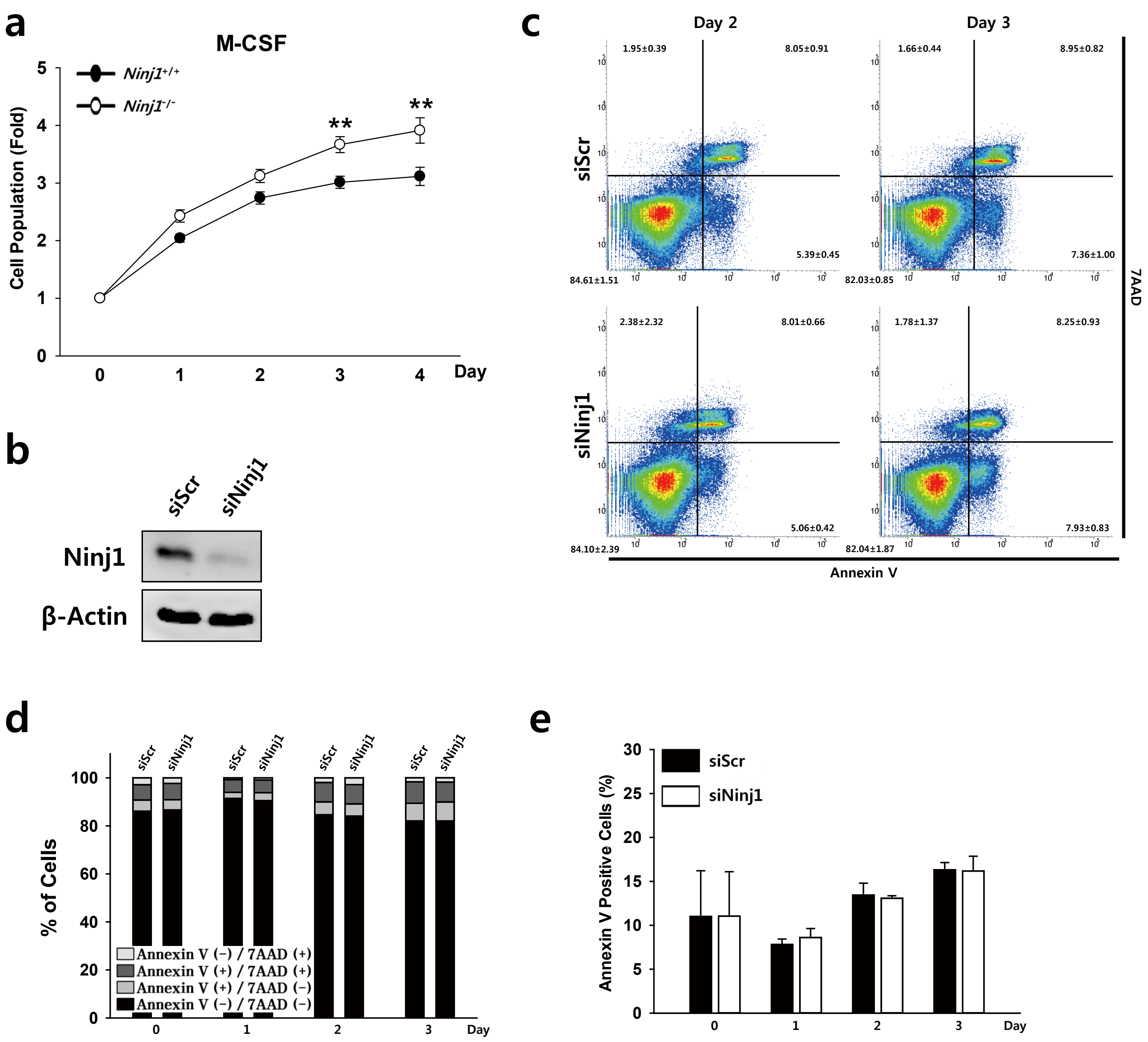

Fig. S6. M-CSF-induced proliferation of BMMs and analysis of TNF-α-induced apoptosis in RAW264.7 cells. (a) BMMs were cultured with M-CSF and relative cell growth, evaluated at indicated days, was shown as mean ± SD (n = 3, triplicate in each experiment), **P<0.01. (b–e) RAW264.7 cells were transfected with siScr or siNinj1 and cultured with TNF-α (10 ng/mL). Cells were harvested and stained with FITC-Annexin V and 7AAD at indicated days followed by FACS analysis. (b) Downregulation of Ninj1 by siRNA was assessed by immunoblot assay at day 1. (c) Representative density plots of days 2 and 3 analyzed by FACS. (d) FACS-determined statistical stacked bars (n = 3). (e) FACS-determined percent frequency of Annexin V^{+} cells. Data are shown as mean ± SD (n = 3).

Figure S7

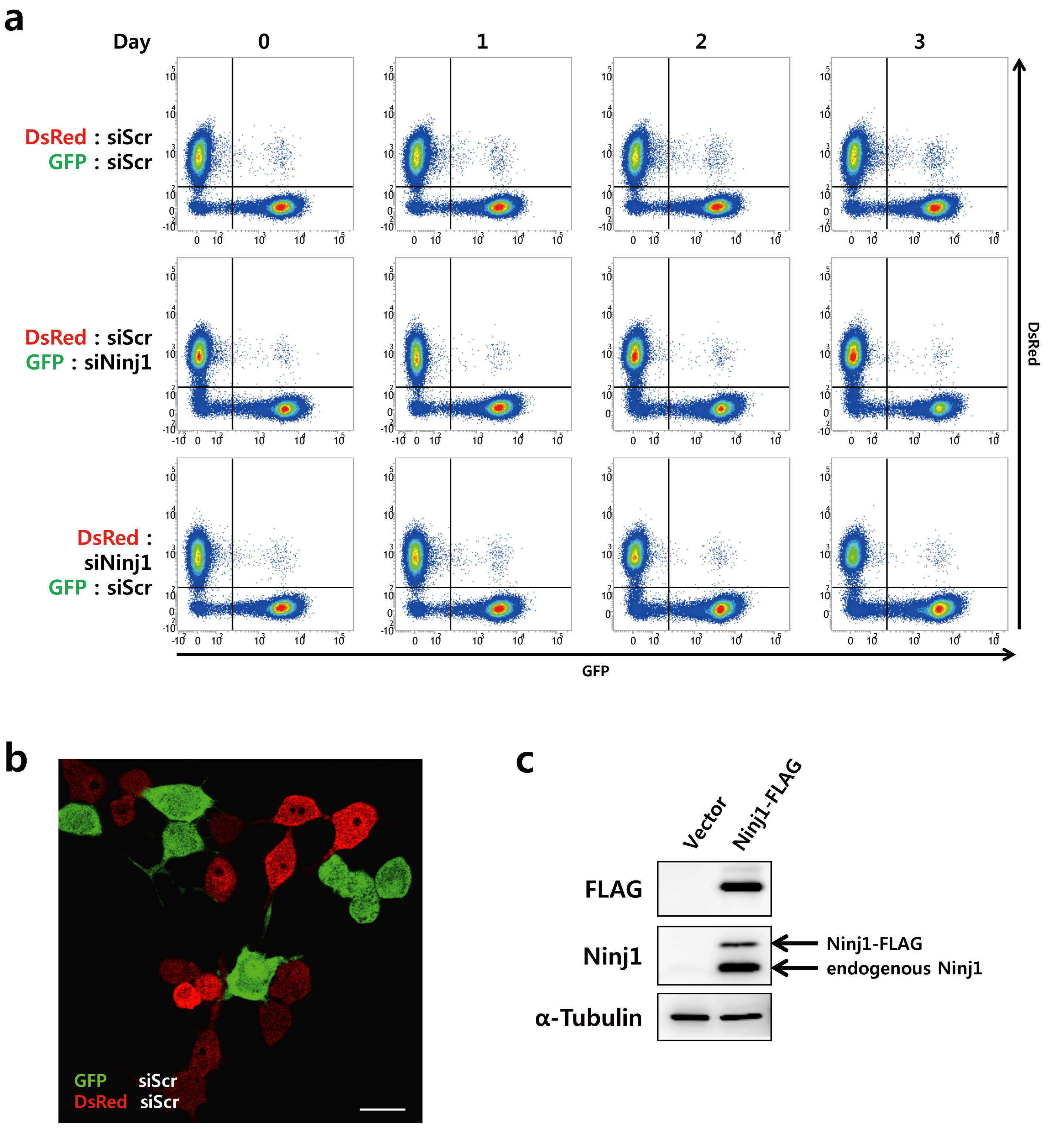

Fig. S7. Mixed culture of GFP$^+$ and DsRed$^+$ RAW264.7 cells and overexpression of Ninj1-FLAG in RAW264.7 cells. (a, b) RAW264.7 cells were stably transduced with GFP or DsRed by retrovirus. GFP$^+$ or DsRed$^+$ RAW264.7 cells were transfected with scrambled siRNA or Ninj1 siRNA as indicated. Equal number of each fluorescence protein labeled cells was co-cultured with RANKL and analyzed by FACS. (a) Representative density plots at indicated days. (b) Representative image of mixed culture. Scale bars, 20 μm. (c) Stable expression of Ninj1-FLAG in RAW264.7 cells was validated by immunoblot assay.

Table S1. Primers and amplification conditions for quantitative RT-PCR.

Gene	Forward (5’ → 3’)	Reverse (5’ → 3’)	Amplicon (bp)	Annealing (℃)
Gapdh	CAGTGCCAGCCTCGTCCCGTAGA	CTGCAAATGGCAGCCCTGGTGAC	95	60
Hprt	GTAATGATCAGTCAACGGGGGAC	CCAGCAAGCTTGCAACCTTAACCA	117	60
Ninj1	TCTTCATTACGGCCTTCGGG	CCCTTAAAGTCTCTGGGCGTT	80	60
Nfatc1	CCCGTCACATTCTGGTCCAT	CAAGTAACCGTGTAGCTGCACAA	145	60
c-Fos	CGGGTTTCAACGCCGACTA	TTGGCACTAGAGACGGACAGA	166	60
Itgb3	GATGACATCGAGCAGGTGAAAGAG	CCGGTCATGGATAGTGATGAGTAG	264	60
Oscar	TCTGCCCCCTATGTGCTATCA	AGGAGCCAGAACCTTCGAAAC	67	60
Calcr	GCCTCCCCATTTACATCTGC	CTCCTCGCCTTCGTTGTTG	68	60
Trap	CGACCATTGTTAGCCACATACG	CACATAGCCCACACCGTTCTC	96	60
Ctsk	AATACGTGCAGCAGAACGGAGGC	CTCGTTCCCCACAGGAATCTCTCTGTAC	137	60
Mmp9	CAGGGAGATGCCCATTTCG	GGGCACCATTTGAGTTTCCA	91	58
c-Src	GGACAGCGGCGGTTTCTACATC	AGCTGCTGCAGGCTGTTGA	57	62
Atp6v0d2	GAAGCTGTCAACATTGCAGA	TCACCGTGATCCTTGCAGAAT	191	60
Dcstamp	CAAGGAACCCAAGGAGTCGT	ACCCAAGTCTCAGACACACTG	101	60
Itgav	GTGTCCGACCACCTCAAGAA	GTGGTGAACTTGGAGCGGA	126	60
Cd44	TGGTGATCAACGGTGGCAAT	GGGGTCTCTGATGGTTCCTTG	118	60
